# Morphology of Rat Hippocampal CA1 Neurons Following Modified Two and Four-Vessels Global Ischemia Models

**DOI:** 10.5812/atr.10240

**Published:** 2013-12-01

**Authors:** Mohammad Ali Atlasi, Homayoun Naderian, Mahdi Noureddini, Esmaeil Fakharian, Abolfazl Azami

**Affiliations:** 1 Anatomical Sciences Research Center, Kashan University of Medical Sciences, Kashan, IR Iran; 2 Physiology Research Center, Kashan University of Medical Sciences, Kashan, IR Iran; 3 Trauma Research Center, Kashan University of Medical Sciences, Kashan, IR Iran

**Keywords:** Hippocampus, Brain Ischemia, Global, Rat

## Abstract

**Background:**

An appropriate animal model of ischemia stroke is essential for evaluation of different therapeutic methods. Two and four-vessel global ischemia models are one of the most common types of transient cerebral ischemia.

**Objectives:**

In this study, the morphology of rat hippocampal CA1 neurons in modified models of two and four-vessel ischemia and reperfusion were evaluated.

**Materials and Methods:**

In this study, 20 Wistar rats were randomly divided into five groups. In group 2 and 3, both common carotid arteries were occluded for 10 minutes in either 3 or 24 hours of reperfusions, respectively. In group 4 and 5, both common carotid and vertebral arteries were occluded for 10 minutes in either 3 or 24 hours of reperfusions, respectively. Group 1 as control, underwent the whole surgery without any arteries occlusion. Hippocampi of the rats in all groups were processed and tissue sections were stained using the Nissl method. The morphology of CA1 neurons were studied under a light microscope and compared different groups.

**Results:**

In all groups ischemic changes were apparently observed in hippocampus CA1 neurons. In two-vessel occlusion model, after 3 and 24 hours of reperfusions, ischemic cells accounted for 14.9% and 23.2%, respectively. In four-vessel occlusion model, after 3 and 24 hours of reperfusions, ischemic cells accounted for 7.6% and 44.9% (P < 0.0001), respectively.

**Conclusions:**

Modified four-vessel occlusion model resulted in significant ischemic changes after 24 hours of reperfusion in CA1 neurons of rat hippocampus.

## 1. Background

Cerebral ischemia/reperfusion (I/R) may happen with high morbidity after shock, brain injury, and cardiac arrest (cardiopulmonary arrest) (1). Cerebral ischemia is a result of insufficient blood supply to a part of the brain, which causes various pathophysiological changes (2). The main event during I/R is the production of reactive oxygen species (ROS), which leads to neuronal death, brain edema and inflammation (3). Transient global ischemia induces neuronal damages specifically in the CA1 region of rats hippocampus (4). Several animal models of cerebral ischemia have been developed to mimic the clinical situation of the humans as accurately as possible. The anatomy of the cerebral vasculature does not grossly differ in rodents and human, therefore rodent models have often been used in animal experiments on cerebral ischemia (5). Rodent models of global ischemia include ligating bilateral common carotid arteries (6-9), increasing intracranial pressure with injecting artificial cerebrospinal fluid (10), and inducing a cardiac arrest by injecting KCL intracardially (11).

Four-vessel occlusion (4VO) model described by Pulsinelli is the most commonly used technique for creation of global ischemia model (12). This technique includes 2-stage surgery within a 24-hour interval and it possibly leads to a more consistent reduction in cerebral blood flow (CBF) and production of preconditioning effects. The two-stage surgery is invasive and technically demanding. The two-vessel occlusion model is easier to perform in a single procedure and is fully reversible (13) and so some researchers have used it for evaluation of neuroprotective agents (14-16).

## 2. Objectives

In the present study, we compared the histopathology of rat hippocampal CA1 neurons in a one-stage approach, 4-vessel and 2-vessel occlusion techniques for making global hemispheric ischemia.

## 3. Materials and Methods

Twenty Wistar rats (200 - 300 g) were used in this experiment. Animal handling was performed in accordance with the rules approved by the local research council at Kashan University of Medical Sciences, Kashan, IR Iran.

### 3.1. Experimental Groups

The animals were randomly divided into five groups, each consisted of four rats. Sixteen animals received 10 minutes of ischemia by two-vessel occlusion (2VO) and four-vessels occlusion (4VO) techniques followed by 3 and 24 hours of reperfusion. Animals with sham operation (control group) underwent the same procedures without the vessel occlusion.

### 3.2. Four-vessel Occlusion Technique

Four-vessel occlusion method was produced with one stage approach. At first eight animals were anaesthetized with ketamine (100 mg/kg) and xylazine (10 mg/kg) intraperitonealy and the vertebral arteries were eletrocoagulated through the alar foramina of the first cervical vertebra. Then a 2.5-cm midline skin incision was made on the frontal aspect of neck and common carotid arteries were separated from vagal nerves, and temporarily occluded by atraumatic microclips for 10 minutes. Reperfusion was introduced by re-infusing of the shed blood by releasing the clamps placed around the carotid arteries.

### 3.3. Two-vessel Occlusion Technique

Eight animals were anaesthetized with ketamine (100 mg/kg) and xylazine (10 mg/kg) intraperitonealy. A 2.5-cm midline skin incision was made on the frontal aspect of neck and temporary occlusion was created by clamping of both common carotid arteries for 10 minutes. The reperfusion phase of 2VO technique was similar to 4VO technique.

### 3.4. Histopathology

Animals were anaesthetized with ketamine (100 mg/kg) and xylazine (10 mg/kg) intraperitonealy and pre-fixation was achieved by trans-cardiac perfusion with 150 mL normal saline followed by 200 mL of 4% paraformaldehyde (PFA) in 0.1 M phosphate buffer. The brains were removed, post-fixed in 4% PFA at 4°C and cut coronally into 3 mm-thick sections including the hippocampal area. For observation under light microscope, the samples were embedded in paraffin and 6 µm coronal sections (one from each five sections) were stained with Nissl method in which the sections were stained with 0.5% cresyl violet, dehydrated through graded alcohols (70, 80, 90, and 100% ×2), placed in xylene, and coverslipped using histomount medium. Finally the slides were studied under the light microscope (Nikon, Germany). By a camera connected to the microscope, the ×100, ×200 and ×400 images of the sections were prepared, and then intact and ischemic cells in CA1 field were counted (17). All data were expressed as mean ± SEM. The statistical test of one-way analysis of variance (ANOVA) with Tukey’s multiple comparison tests was used for comparison of all groups. The P value of < 0.05 was considered as statistically significant.

## 4. Results

**Figure 1.**
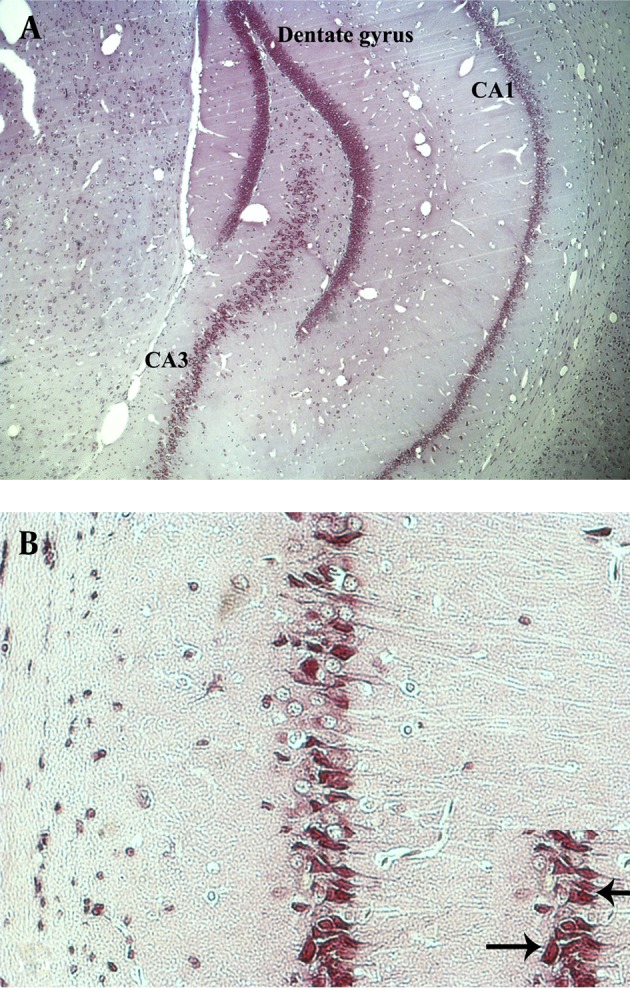
Photomicrographs Show the Morphology of Hippocampus by Nissl Staining Method (A ×4, B ×400). Arrows Show the Ischemic Cells in the Hippocampal CA1 Region (Panel in B)

The CA1 pyramidal cells of rat hippocampus after 2VO or 4VO procedure of ischemia/reperfusion, exhibited morphological alterations consistent with a degenerative process (Figure 1), and the mean number of damaged cells in both methods varied according to the reperfusion period and type of technique. In the control group, most of the pyramidal neurons had a round or oval nucleus, located in the center of perikarion that are surrounded by pale cytoplasm. 

In comparison with the control animals, the rats submitted to global transitory ischemia followed by 3 hour-reperfusion in both 2VO and 4VO techniques showed slight morphological alterations in most of their neurons. In animals submitted to 10 minutes of ischemia and 24 hours of reperfusion in 2VO group, the ischemic changes were more evident than the group with 3 hour-reperfusion. These alterations were evidently more severe in 4VO group after 24 hours reperfusion. The neuronal changes were triangular in shape mostly exhibiting a dark staining due to condensation of cytoplasm and karyoplasms (Figure 1B). 

**Figure 2.**
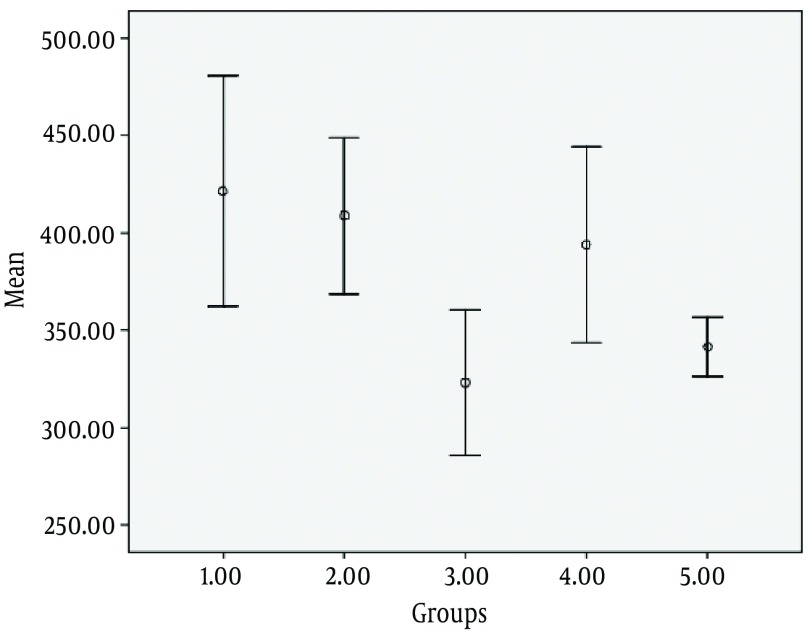
Average Number of CA1 Neurons in Different Groups 1. Control (Sham) group 2. Two-vessel occlusion + 3 hours of reperfusion 3. Two-vessel occlusion + 24 hours of reperfusion 4. Four-vessel occlusion + 3 hours of reperfusion 5. Four-vessel occlusion + 24 hours of reperfusion.

As shown in Figure 2, the mean number of CA1 neurons in the control group was 4213.1 ± 1261. In 2VO technique, after 10 minutes of carotid artery occlusion and 3 hour-reperfusion, the mean number of CA1 neurons was 4088.3 ± 803 (P < 0.9), and after a 24 hour-reperfusion this amount was 3232.1 ± 890 (P < 0.003). In 4VO technique, after a 3 hour-reperfusion, the average number of pyramidal neurons was 3939.5 ± 599 (P < 0.9) and after a 24 hour-reperfusion this amount was 3416 ± 324 (P < 0.03). 

**Figure 3.**
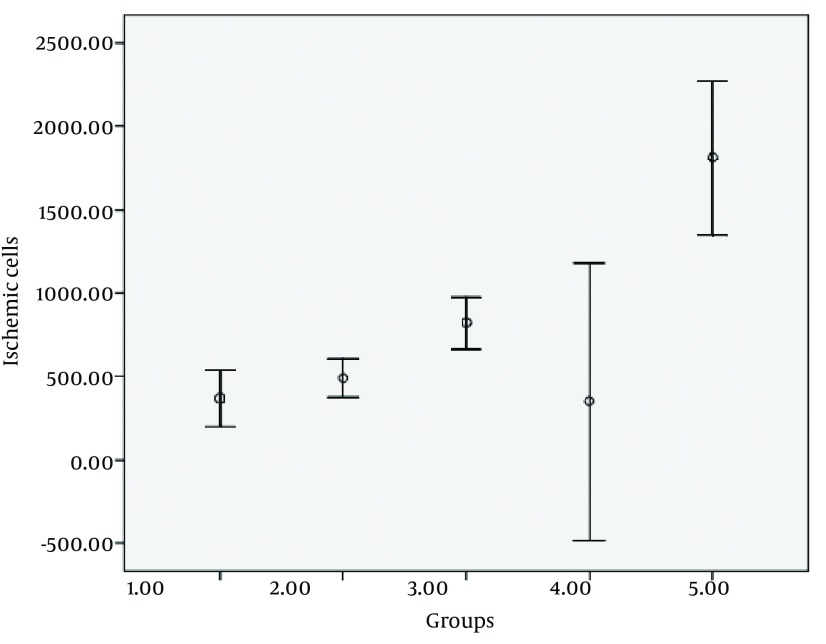
Average of CA1 Ischemic Neurons in Different Groups 1. Control (Sham) group 2. Two vessel occlusion + 3 hours of reperfusion 3. Two vessel occlusion + 24 hours of reperfusion 4. Four vessel occlusion + 3 hours of reperfusion 5. Four vessel occlusion + 24 hours of reperfusion

As shown in Figure 3, in control group, the mean number of ischemic cells in CA1 field was 369.45 ± 358 (8%). In 2VO technique, following 10 minutes carotid artery occlusion and 3 hours of reperfusion, the mean number of ischemic cells in this area were 492.39 ± 236 (14.9%; P < 0.9) and after 24 hours of reperfusion this amount was 822.46 ± 37 (23.2%; P < 0.1). In 4VO technique, after 3 hours of reperfusion the average number of damaged cells was 352 ± 995 (7/6%; P < 1) and following a 24 hour-reperfusion this amount was 1813 ± 98 (44.9%; P < 0.0001). 

## 5. Discussion

Transient global ischemia leads to neuronal damage and cell death (14). This study showed that the number of CA1 subfield damaged cells following an ischemia/reperfusion is related to the type of vessel occlusion and duration of reperfusion.

The four-vessel occlusion model is widely used by investigators for production of transient cerebral ischemia and studying the effectiveness of potential neuroprotective agents (12, 18, 19). This model is a two-stage surgery with permanent occlusion of the vertebral arteries on the first day followed by transient occlusion of the common carotid arteries on the following day. This model has been modified over the years. In our study the 4-vessel occlusion technique was carried out in one stage approach through a minimally invasive surgical procedure. Originally four-vessel occlusion technique was performed with two stage procedures (12) which may lead to be a risk factor of the animal mortality and occurrence of collateral circulation and preconditioning effect due the 24-hour interval between two stages of surgery. Yamaguchi et al. showed consistent neurological deficits and morphological changes in CA1, CA2, CA3, and dentate gyrus with modification of 4-vessel occlusion technique to one stage approach (20). In the present study in the 4-vessel occlusion technique, the number of CA1 ischemic cells after 10 minutes of ischemia and 24 hours of reperfusion were significantly increased. The difference between our study and Yamaguchi et al. was in the approach for the exposure of the vertebral arteries. Yamaguchi et al. used anterior approach for finding these arteries but similar to Pulsinelli et al. (12), we find out vertebral arteries via an incision behind the occipital bone directly overlying the first two cervical vertebrae.

In our study, the time of ischemia was 10 minutes. The pyramidal cells of CA1 subfield of hippocampus showed a significant degree of vulnerability to a transient ischemic damage of short duration (≤ 15 min) in four-vessel occlusion (21, 22). It was reported that the optimum time of effective ischemia was 10 minutes in the four-vessel occlusion rat model (7), whilst McBean et al. used two-vessel occlusion model, and found that in the anterior and posterior neocortex, thalamus, striatum and cerebellum this time was consistent with four-vessel occlusion model but damage to the hippocampal CA cells was not complete and bilateral arterial occlusion in 12 minutes enhanced the reproducibility of neuropathological scores. In our study, it seems that the lack of damage to the neurons increased in two-vessel occlusion model which is related to the vessels occlusion time, and despite of the two-vessel occlusion technique, it was shown that in four-vessel occlusion technique following 24 hours of reperfusion, ischemic cells increased significantly in CA1 field. There are evidences that cell death (apoptosis) occurs in the CA1 field of rat hippocampus after transient ischemia followed by early reperfusion times (21, 22). It was shown that reperfusion after 3 hours resulted in slight ultrastructural alterations in involved CA1 neurons, and after 12 hours pathway of cell death pathway has been changed from necrosis to apoptosis. Reperfusion after transient ischemia accelerated the onset of morphological changes (22). Reperfusion effects often include activation of oxidant production by cytoplasmic and mitochondrial O_2_-dependent enzymes and resulted to the destruction of cellular proteins and membranes and finally cell death (23, 24). Although the number of neurons in two and four-vessel occlusion groups after 24 hours of reperfusion was significantly decreased, but it seems that for a better estimation of total cell number in hippocampus, it is necessary to apply new stereological methods such as optical fractionator design (25).

In conclusion, although the original versions of two-vessel and four-vessel occlusion models are the most commonly used models of global cerebral ischemia, the modifications in four-vessel occlusion model described in this paper offer the advantages of morphological alterations of CA1 neurons toward the damage.
